# Reducing the Spectral Radius of a Torus Network by Link Removal

**DOI:** 10.1371/journal.pone.0155580

**Published:** 2016-05-12

**Authors:** Xiaofan Yang, Pengdeng Li, Lu-Xing Yang, Yingbo Wu

**Affiliations:** School of Software Engineering, Chongqing University, Chongqing, China; Southwest University, CHINA

## Abstract

The optimal link removal (OLR) problem aims at removing a given number of links of a network so that the spectral radius of the residue network obtained by removing the links from the network attains the minimum. Torus networks are a class of regular networks that have witnessed widespread applications. This paper addresses three subproblems of the OLR problem for torus networks, where two or three or four edges are removed. For either of the three subproblems, a link-removing scheme is described. Exhaustive searches show that, for small-sized tori, each of the proposed schemes produces an optimal solution to the corresponding subproblem. Monte-Carlo simulations demonstrate that, for medium-sized tori, each of the three schemes produces a solution to the corresponding subproblem, which is optimal when compared to a large set of randomly produced link-removing schemes. Consequently, it is speculated that each of the three schemes produces an optimal solution to the corresponding subproblem for all torus networks. The set of links produced by each of our schemes is evenly distributed over a network, which may be a common feature of an optimal solution to the OLR problem for regular networks.

## 1 Introduction

The epidemic modeling is recognized as an effective approach to the understanding of propagation process of objects over a network [1, 2]. For instance, epidemic models help understand the prevalence of malware [3–9]. The speed and extent of spread of an epidemic over a network depend largely on the structure of the network; whether the epidemic tends to extinction is determined by the spectral radius of the network [8, 10, 11]. As a smaller spectral radius contributes to the containment of an undesirable epidemic, a natural option to mitigate the negative effect of the epidemic is to reduce the spectral radius of the underlying network.

Preciado and Jadbabaie [12] suggested to remove links from a network so as to reduce its spectral radius. The optimal link removal (OLR) problem aims at removing a given number of links of a network so that the spectral radius of the residue network obtained by removing the links from the network attains the minimum. As the OLR problem is NP-hard [13], it is much unlikely that there exist an efficient algorithm for solving the problem. As a result, multifarious heuristics for solving the problem have been proposed [13–19]. In most situations, however, these heuristics produce non-optimal solutions.

Torus networks are a class of regular networks [20]. Due to the superiority in unicast routing [21–26], multicast routing [27] and fault tolerance [28], torus networks frequently serve as the underlying interconnection network of a multicomputer system. To our knowledge, there is no report in literature on the OLR problem for torus networks.

This paper addresses three subproblems of the OLR for two-dimensional torus networks, where two or three or four edges are removed, respectively. For either of the three subproblems, a link-removing scheme is described. Exhaustive searches show that, for small-sized tori, each of the proposed schemes produces an optimal solution to the corresponding subproblem. Monte-Carlo simulations demonstates that, for medium-sized tori, each of the three schemes produces a solution to the corresponding subproblem, which is optimal when compared to 10,000 randomly produced sets of links. Consequently, it is speculated that each of the three schemes produces an optimal solution to the corresponding subproblem for all torus networks. The set of links produced by each of our schemes is evenly distributed over a network, which may be a common feature of an optimal solution to the OLR problem for regular networks.

The remaining materials are organized in this fashion: The preliminary knowledge is provided in Section 2. Section 3 presents the main results of this work. Finally, Section 4 outlines this work.

## 2 Preliminaries

For fundamental knowledge on the spectral radius of a network, see Ref. [29]. The optimal link removal (OLR) problem is formulated as follows: Given a network *G* = (*V*, *E*) and a positive integer *k*, find a set of *k* links of *G* so that the spectral radius of the residue network obtained by removing the links from the network attains the minimum.

An *N* × *N* torus network, denoted **T**_*N*_, is a network with node set *V* = {(*i*, *j*): *i*, *j* = 0, 1,…,*N* − 1}, which has the following two kinds of links:

- {(*i*, *j*), ((*i* + 1) mod *N*, *j*)}, which is abbreviated as (*i*, *j*) →.
- {(*i*, *j*), (*i*, (*j* + 1) mod *N*)}, which is abbreviated as (*i*, *j*) ↑.

**Fig 1:**
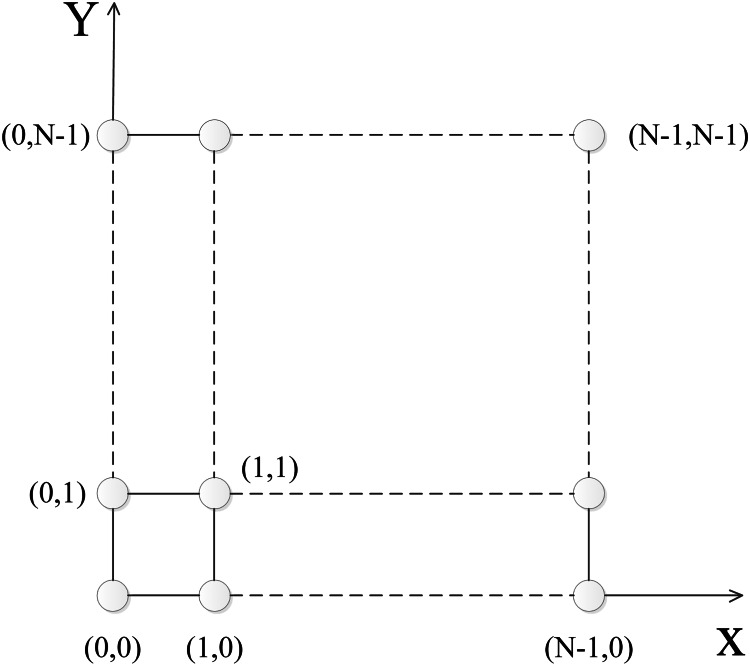
The diagram of T_*N*_.

Fig 1 gives a schematic diagram of **T**_*N*_.

## 3 Main results

This section considers the optimal scheme of removing two or three or four links from **T**_*N*_.

### 3.1 Removing two links

Let us consider a subproblem of OLR problem, where the network is torus, and two edges are removed. Denote the subproblem by OLR-T2.

In view of the symmetry of **T**_*N*_, we may assume that one of the two links to be removed from **T**_*N*_ is *e*_1_ = (0, 0) →. Now, let us choose the second link *e*_2_ to be removed from **T**_*N*_ in the following way.

If *N* is even, then

$$\begin{array}{c} e_{2}=\left(\frac{N}{2},\frac{N}{2}\right)\to . \end{array}$$

If *N* is odd, then

$$\begin{array}{c} e_{2}=\left(\frac{N+1}{2},\frac{N-1}{2}\right)↑. \end{array}$$

**Fig 2:**
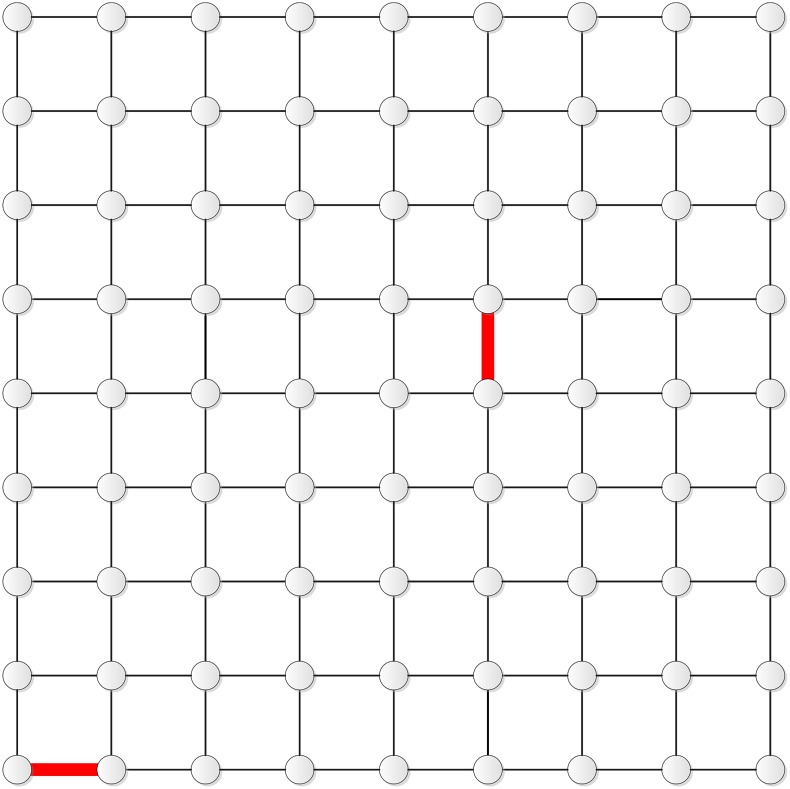
The two edges in T_9_.

**Fig 3:**
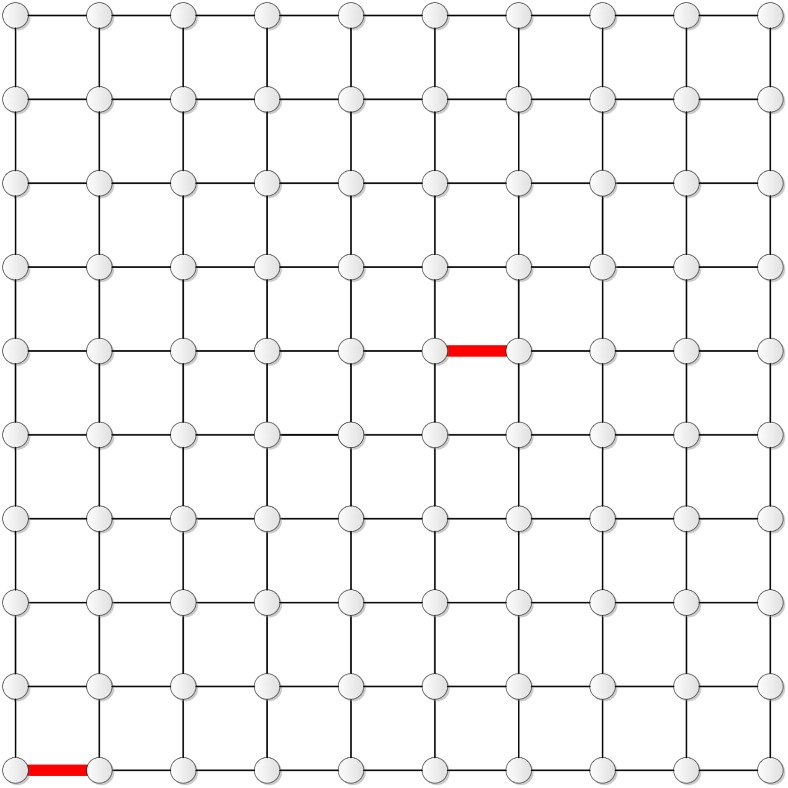
The two edges in T_10_.

Figs 2 and 3 show the two edges determined by this scheme for **T**_9_ and **T**_10_, respectively.

**Fig 4:**
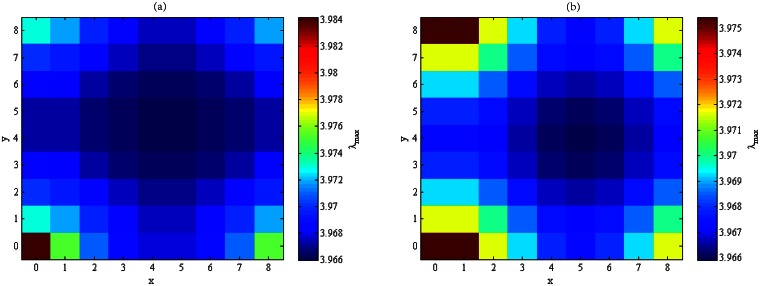
The spectral radii of all residue networks of T_9_ obtained by removing (a) → *e*_2_, or (b) ↑ *e*_2_.

**Fig 5:**
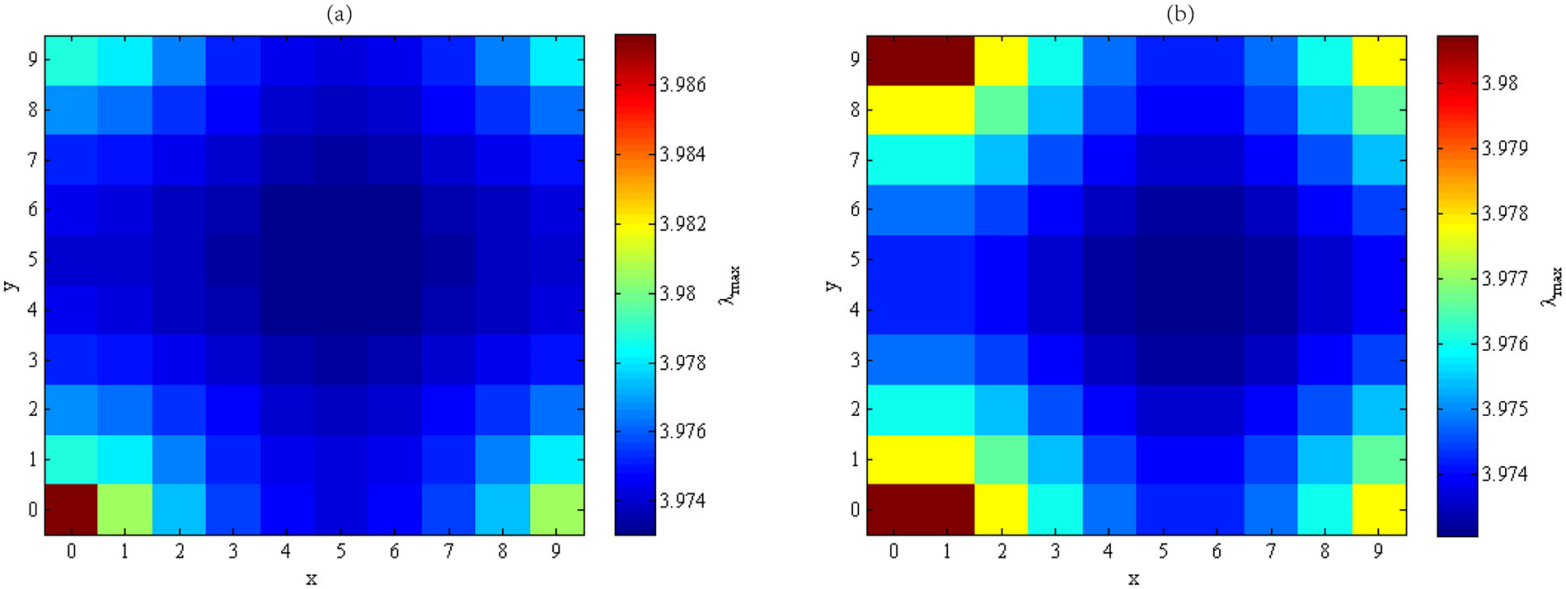
The spectral radii of all residue networks of T_10_ obtained by removing (a) → *e*_2_, or (b) ↑ *e*_2_.

Exhaustive search gives the spectral radii of all residue networks obtained by removing two links from **T**_*N*_, 3 ≤ *N* ≤ 20, and Figs 4 and 5 exhibit the spectral radii of all residue networks of **T**_9_ and **T**_10_, respectively. It can be seen that the larger the distance between the two links, the smaller the spectral radius of the residue network. At the extreme, the spectral radius of the residue network **T**_*N*_ − {*e*_1_, *e*_2_} attains the minimum among all residue networks obtained by removing two links from **T**_*N*_. That is, the proposed scheme produces an optimal solution to the OLR-T2 problem.

**Fig 6:**
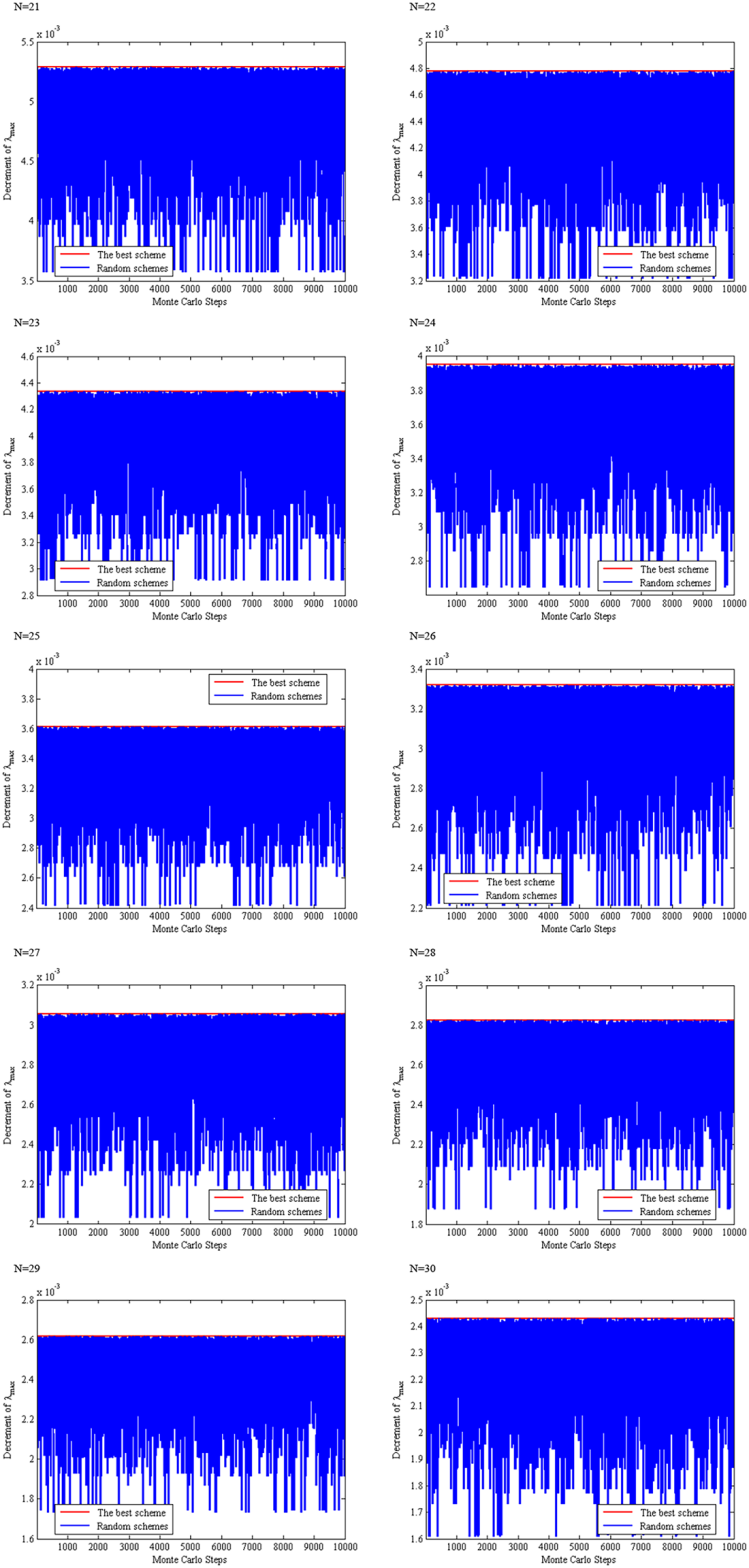
A comparison between the proposed scheme and 10,000 randomly produced schemes.

For 21 ≤ *N* ≤ 30, the above scheme is compared with 10,000 randomly produced schemes of removing two links in terms of the spectral radius of the residue network, see Fig 6. Clearly, the proposed scheme produces an optimal solution as compared to the 10,000 schemes. Therefore, we propose the following conjecture.

**Conjecture 1.** *For N* ≥ 3, *the proposed scheme produces an optimal solution to the OLR-T2 problem*.

### 3.2 Removing three links

Let us consider a subproblem of OLR problem, where the network is torus, and three links are removed. Denote the subproblem by OLR-T3.

In view of the symmetry of **T**_*N*_, we may assume that one of the three links to be removed from **T**_*N*_ is *e*_1_ = (0, 0) →. Now, let us choose the other two links, *e*_2_ and *e*_3_, to be removed from **T**_*N*_ in the following way.

If *N* ≡ 0 mod 3, then

$$\begin{array}{c} e_{2}=\left(\frac{N}{3},\frac{N}{3}\right)\to ,\;e_{3}=\left(\frac{2N}{3},\frac{2N}{3}\right)\to . \end{array}$$

If *N* ≡ 1 mod 3, then

$$\begin{array}{c} e_{2}=\left(\left\lfloor \frac{N+1}{3}\right\rfloor ,\frac{2N+1}{3}-\left\lfloor \frac{N+1}{3}\right\rfloor \right)\to , \end{array}$$

$$\begin{array}{c} e_{3}=\left(\left\lfloor \frac{N+1}{3}\right\rfloor +\frac{N+2}{3},N-1-\left\lfloor \frac{N+1}{3}\right\rfloor \right)↑. \end{array}$$

If *N* ≡ 2 mod 3 and *N* > 5, then

$$\begin{array}{c} e_{2}=\left(\left\lfloor \frac{N+2}{3}\right\rfloor ,\frac{2N+2}{3}-\left\lfloor \frac{N+2}{3}\right\rfloor \right)\to , \end{array}$$

$$\begin{array}{c} e_{3}=\left(\left\lfloor \frac{N+2}{3}\right\rfloor +\frac{N+1}{3},N-\left\lfloor \frac{N+2}{3}\right\rfloor \right)↑. \end{array}$$

If *N* = 5, then

$$\begin{array}{c} e_{2}=\left(0,2\right)\to ,\;e_{3}=\left(3,3\right)↑. \end{array}$$

**Fig 7:**
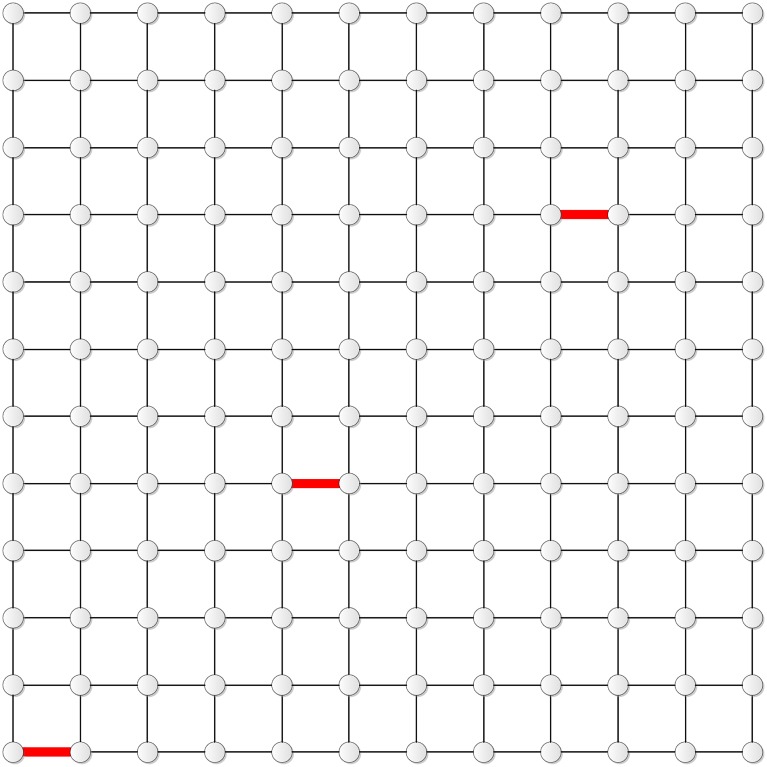
The distribution of the three edges in T_12_.

**Fig 8:**
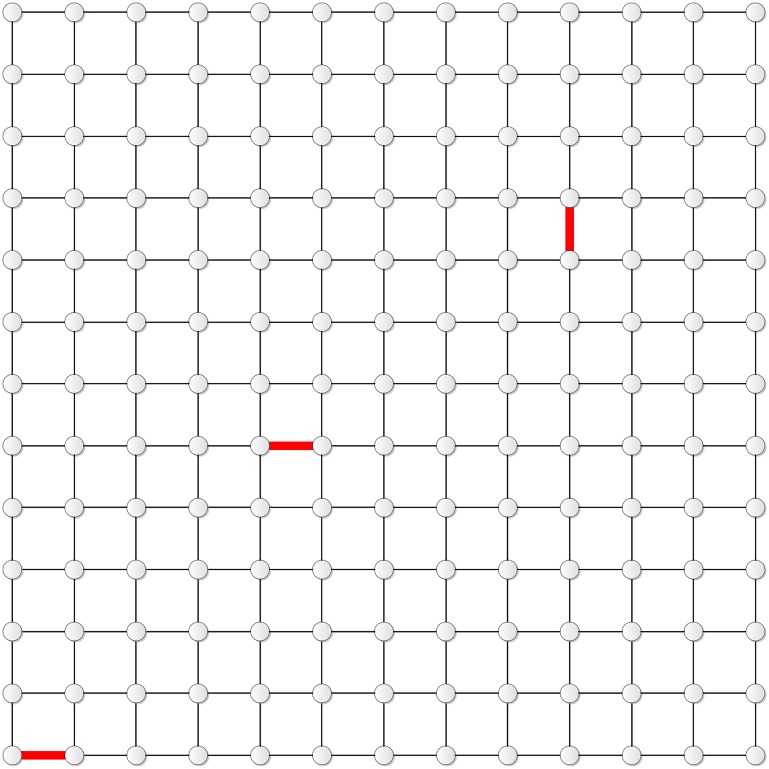
The distribution of the three edges in T_13_.

**Fig 9:**
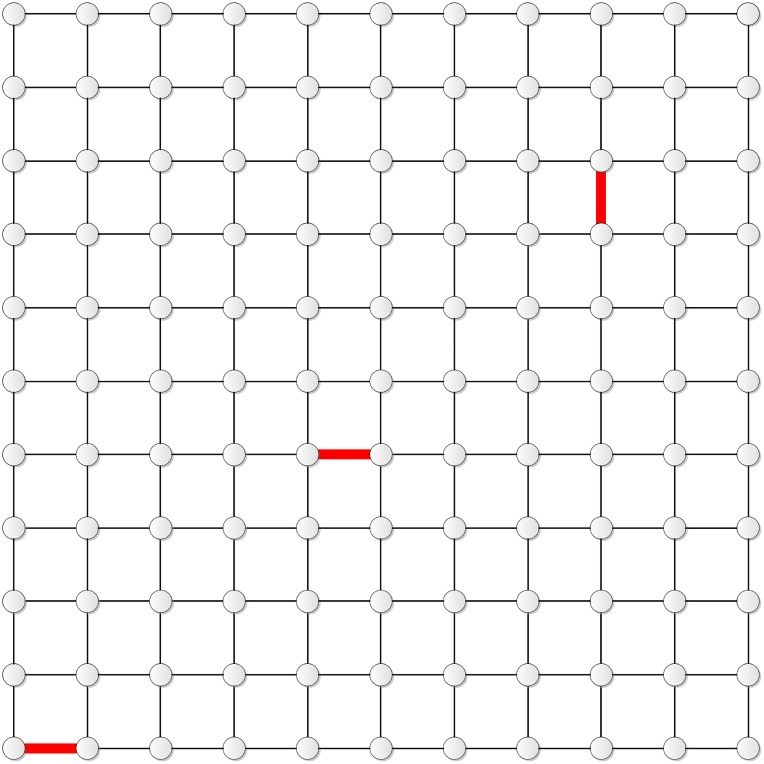
The distribution of the three edges in T_11_.

Figs 7–9 show the distribution of the three edges in **T**_12_, **T**_13_, and **T**_11_, respectively.

For 3 ≤ *N* ≤ 20, exhaustive search shows that the spectral radius of the residue network **T**_*N*_ − {*e*_1_, *e*_2_, *e*_3_} attains the minimum among all residue networks obtained by removing three links from **T**_*N*_. That is, the proposed scheme produces an optimal solution to the OLR-T3 problem.

**Fig 10:**
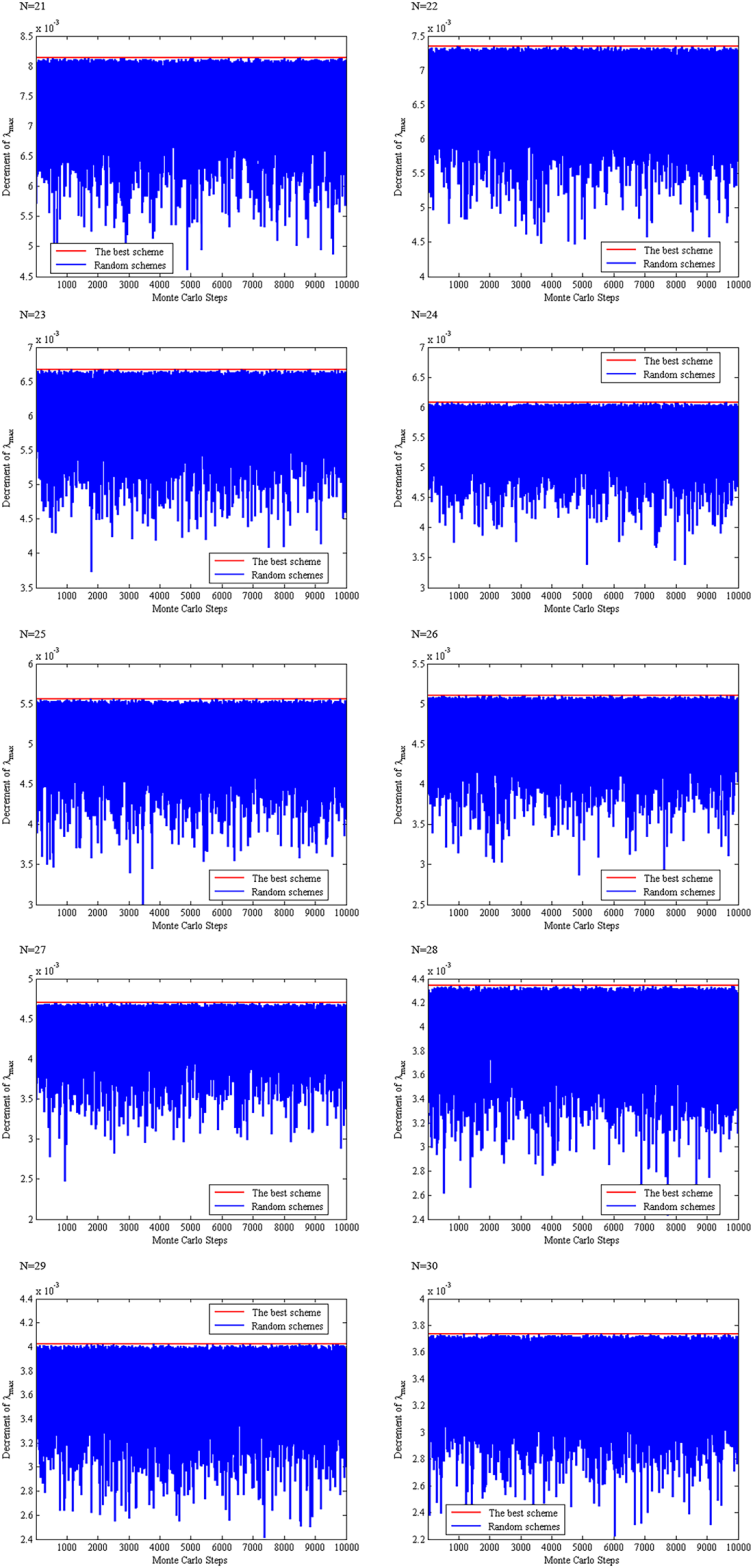
A comparison between our proposed scheme and 10,000 randomly produced schemes.

For 21 ≤ *N* ≤ 30, the above scheme of choosing three links is compared with 10000 randomly produced schemes of choosing three links with respect to the spectral radius of the residue network obtained by removing the three chosen links from **T**_*N*_, see Fig 10. It can be seen from this figure that the proposed scheme produces a residue network with minimum spectral radius among the 10001 schemes (the minimum spectral radius as compared with the 10,000 randomly produced schemes). Therefore, we propose the following conjecture.

**Conjecture 2.** *For N* ≥ 3, *the proposed scheme produces an optimal solution to the OLR-T3 problem*.

### 3.3 Removing four links

Let us consider a subproblem of OLR problem, where the network is torus, and four edges are removed. Denote the subproblem by OLR-T4.

In view of the symmetry of **T**_*N*_, we may assume that one of the four links to be removed from **T**_*N*_ is *e*_1_ = (0, 0) →. Now, let us choose the other three links, *e*_2_, *e*_3_ and *e*_4_, to be removed from **T**_*N*_ in the following way.

If *N* is even, then

$$\begin{array}{c} e_{2}=\left(0,\frac{N}{2}\right)\to , \end{array}$$

$$\begin{array}{c} e_{3}=\left(\frac{N}{2},\left\lfloor \frac{2N-4\left\lfloor \frac{N}{4}\right\rfloor }{4}\right\rfloor \right)\to , \end{array}$$

$$\begin{array}{c} e_{4}=\left(\frac{N}{2},\left\lfloor \frac{4N-4\left\lfloor \frac{N}{4}\right\rfloor }{4}\right\rfloor \right)\to . \end{array}$$

If *N* is odd and *N* > 3, then

$$\begin{array}{c} e_{2}=\left(\left\lfloor \frac{2N-4\left\lfloor \frac{N}{4}\right\rfloor }{4}\right\rfloor ,\left\lfloor \frac{N}{2}\right\rfloor \right)↑, \end{array}$$

$$\begin{array}{c} e_{3}=\left(\frac{N+1}{2},0\right)↑, \end{array}$$

$$\begin{array}{c} e_{4}=\left(\left\lfloor \frac{2N-4\left\lfloor \frac{N}{4}\right\rfloor }{4}\right\rfloor +\left\lfloor \frac{N}{2}\right\rfloor ,N-\left\lfloor \frac{N}{2}\right\rfloor \right)\to . \end{array}$$

If *N* = 3, then

$$\begin{array}{c} e_{2}=\left(2,0\right)↑,\;e_{3}=\left(0,1\right)\to ,\;\;e_{4}=\left(0,2\right)\to . \end{array}$$

**Fig 11:**
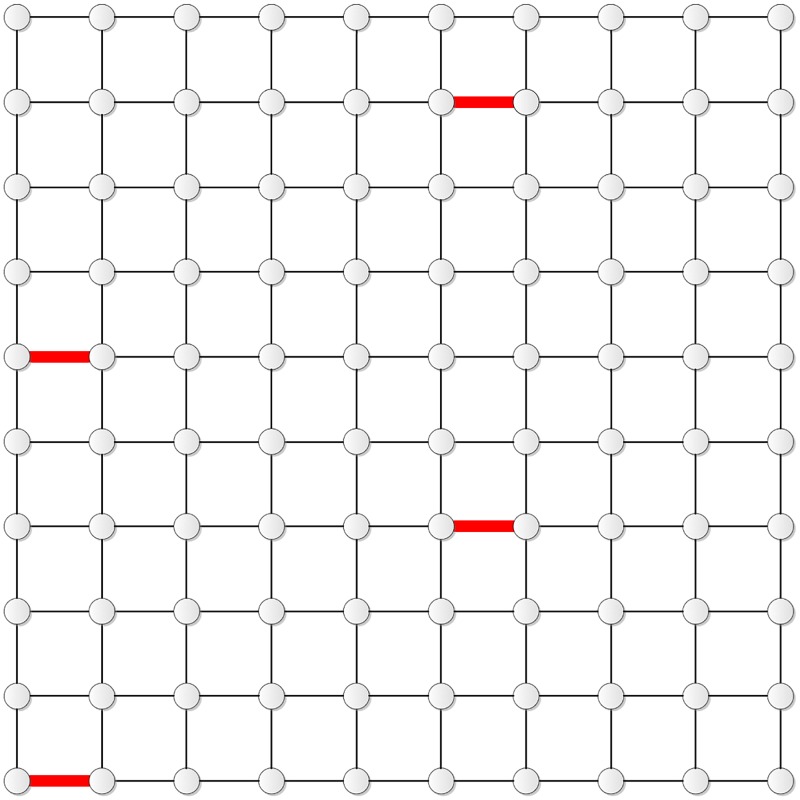
The distribution of the four edges in T_10_.

**Fig 12:**
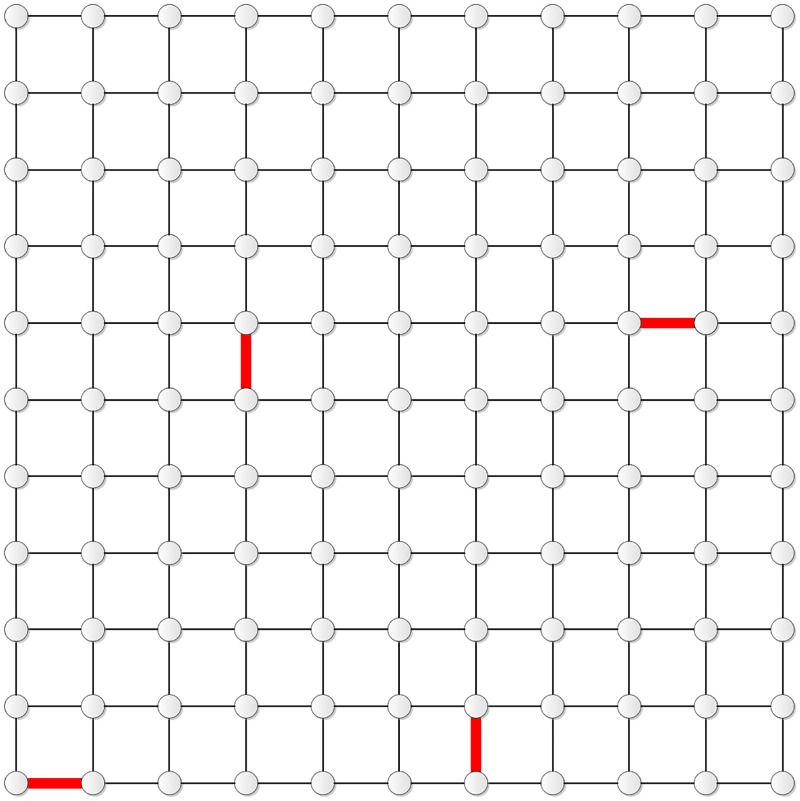
The distribution of the four edges in T_11_.

Figs 11 and 12 show the distribution of the four edges in **T**_10_ and **T**_11_, respectively.

For 3 ≤ *N* ≤ 11, exhaustive search shows that the spectral radius of the residue network **T**_*N*_−{*e*_1_, *e*_2_, *e*_3_, *e*_4_} attains the minimum among all residue networks obtained by removing four links from **T**_*N*_. That is, the proposed scheme produces an optimal solution to the OLR-T4 problem.

**Fig 13:**
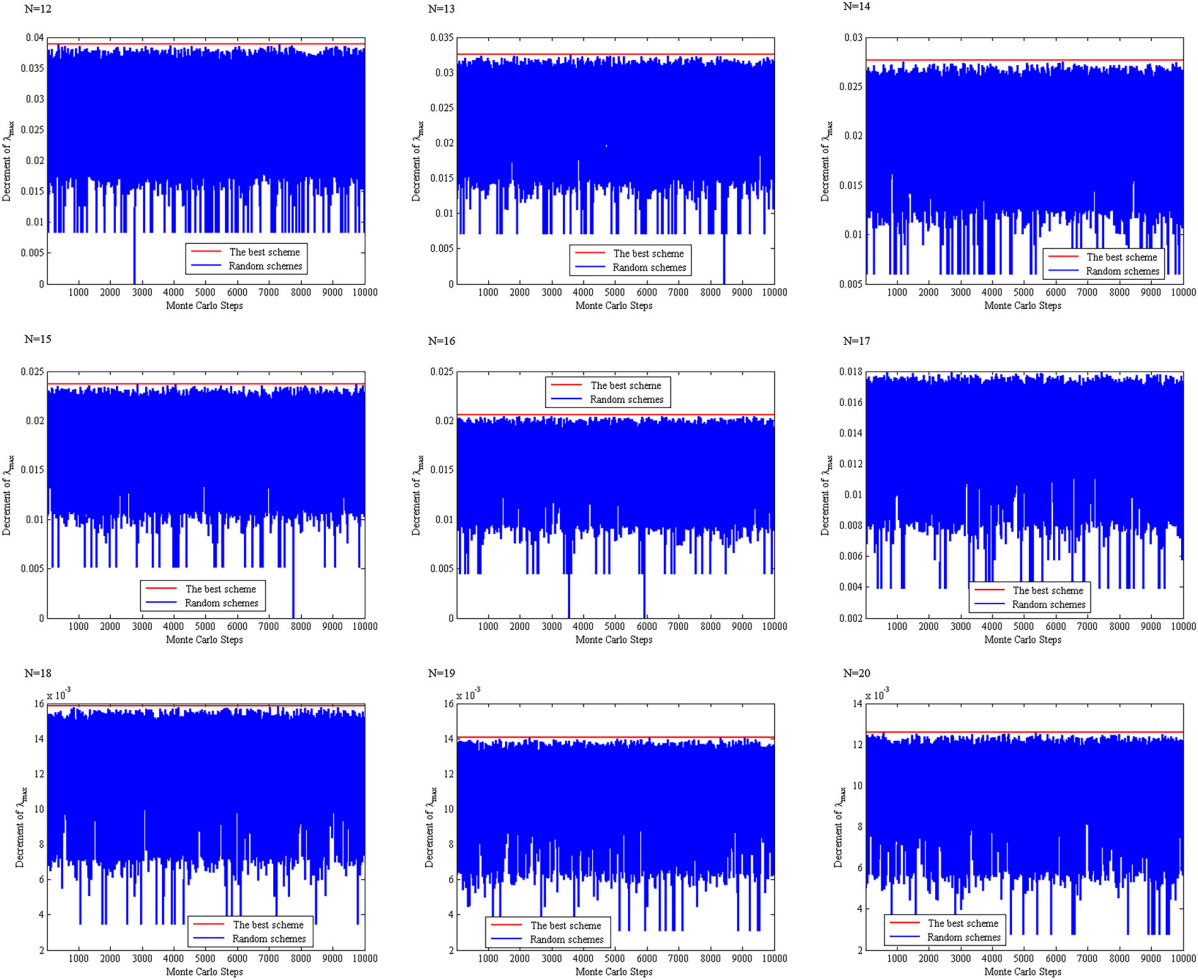
A comparison between our proposed scheme and 10,000 randomly produced schemes.

For 12 ≤ *N* ≤ 20, the above scheme of choosing four links is compared with 10,000 randomly produced schemes of choosing three links with respect to the spectral radius of the residue network obtained by removing the four chosen links from **T**_*N*_, see Fig 13. It can be seen from this figure that the proposed scheme produces a residue network with minimum spectral radius among the 10,001 schemes (the minimum spectral radius as compared with the 10,000 randomly produced schemes). Therefore, we propose the following conjecture.

**Conjecture 3.** *For N* ≥ 3, *the proposed scheme produces an optimal solution to the OLR-T4 problem*.

## 4 Conclusions

In this paper, we have studied the problem of how to delete two, three or four links from an *N* × *N* torus so that the spectral raidus of the residue network is minimized. Given the number of links to be removed, a scheme of removing links has been presented. For smaller *N*, exhaustive search shows that the proposed scheme is optimal among all possibe schemes, because it produces a residue network with minimum spectral radius. For medium-sized *N*, Monte-Carlo experiment shows that the proposed scheme is optimal among 10,000 randomly produced schemes. We guess that the proposed scheme is optimal for all *N* and among all schemes. From the distribution of the links determined by our schemes, we guess that an optimal scheme tends to remove a set evenly distributed set of edges.

In our opinion, similar work should be done for other kinds of regular networks such as the bijective connection networks [30–32].
